# Isolation of Microorganisms Using Sub-Micrometer Constrictions

**DOI:** 10.1371/journal.pone.0101429

**Published:** 2014-06-30

**Authors:** Nil Tandogan, Pegah N. Abadian, Slava Epstein, Yoshiteru Aoi, Edgar D. Goluch

**Affiliations:** 1 Department of Chemical Engineering, Northeastern University, Boston, Massachusetts, United States of America; 2 Department of Biology, Northeastern University, Boston, Massachusetts, United States of America; 3 Institute for Sustainable Science and Development, Hiroshima University, Hiroshima, Japan; University of Illinois at Chicago, United States of America

## Abstract

We present an automated method for isolating pure bacterial cultures from samples containing multiple species that exploits the cell's own physiology to perform the separation. Cells compete to reach a chamber containing nutrients via a constriction whose cross-sectional area only permits a single cell to enter, thereby blocking the opening and preventing other cells from entering. The winning cell divides across the constriction and its progeny populate the chamber. The devices are passive and require no user interaction to perform their function. Device fabrication begins with the creation of a master mold that contains the desired constriction and chamber features. Replica molding is used to create patterned polymer chips from the master, which are bonded to glass microscope cover slips to create the constrictions. We tested constriction geometries ranging from 500 nanometers to 5 micrometers in width, 600 to 950 nanometers in height, and 10 to 40 micrometers in length. The devices were used to successfully isolate a pure *Pseudomonas aeruginosa* culture from a mixture that also contained *Escherichia coli*. We demonstrated that individual strains of the same species can be separated out from mixtures using red and green fluorescently-labeled *E. coli*. We also used the devices to isolate individual environmental species. *Roseobacter sp*. was separated from another marine species, *Psychroserpens sp*.

## Introduction

It is widely recognized that the overwhelming majority of microorganisms cannot grow in artificial media that is prepared in laboratories. This issue was first realized over a century ago when the direct bacterial counts from environmental samples did not correlate with the number of resulting colony forming units (CFUs), a phenomenon known as “great plate count anomaly” [1]–[3]. The problem is not only that very few colonies grow on the plates, but also that only a few species form colonies when compared to direct individual cell analysis of environmental samples. Cultivation-independent approaches, such as rRNA sequencing, have revealed that there remains an immense number of uncultivated species in nature [4], [5]. Thus, the vast complexity of the microbial world is still mostly unexplored and not understood.

Since then, there have been significant efforts made to overcome these limitations in cultivation. Some endeavors consist of modifications to conventional methods, such as changes in the medium composition (or concentration), gelling agents, and incubation time [3], [6]–[15]. Another approach is incubation of microbial cells in their natural environment using membrane bound devices. As shown by several studies, this approach allows the supply of necessary growth factors from native habitats to devices, which then stimulate the growth of uncultured microorganisms [1], [16]–[23]. Given the large number of unexplored species, high-throughput is also an important aspect of cultivation methodologies. This is usually accomplished by miniaturization of the growth compartment. One example is gel micro-droplet technology, which enables high-throughput cultivation and can be applied for high-throughput screening as well [16]. In this respect, microfabrication technologies have remarkable potential to revolutionize microbial cultivation [24]. One such example is the miniaturized cultivation chip, or million well chip, which has one million individual micro-scale growth compartments on a single device [25]. In spite of these advances, further innovations in cultivation technology are critical to increase the number of cultivable species. These advances will elucidate the underlying factors of many diseases, lead to drug discovery, and reveal the missing pieces of the microbial world [26].

In a broad sense, cultivation of microorganisms in pure culture principally requires the following three steps: i) isolation of individual cells from the other microbial cells, ii) inoculation of the single cell into a culture medium, iii) incubation to let the cell propagate, followed by detection and collection of the biomass. We developed a micro- and nano-fabricated device, which allows the above three steps to be performed automatically. It is used to capture single bacterial cells from a mixture of species and produce pure cultures from those cells. The device can be easily placed *in situ* to capture a single cell type, such as an uncultivable species. By having the device located in the cell's natural environment, essential, unidentified growth factors can diffuse into the food chamber and allow a single cell type to proliferate and form a large population of clones, which can be later removed for analysis.

**Figure 1:**
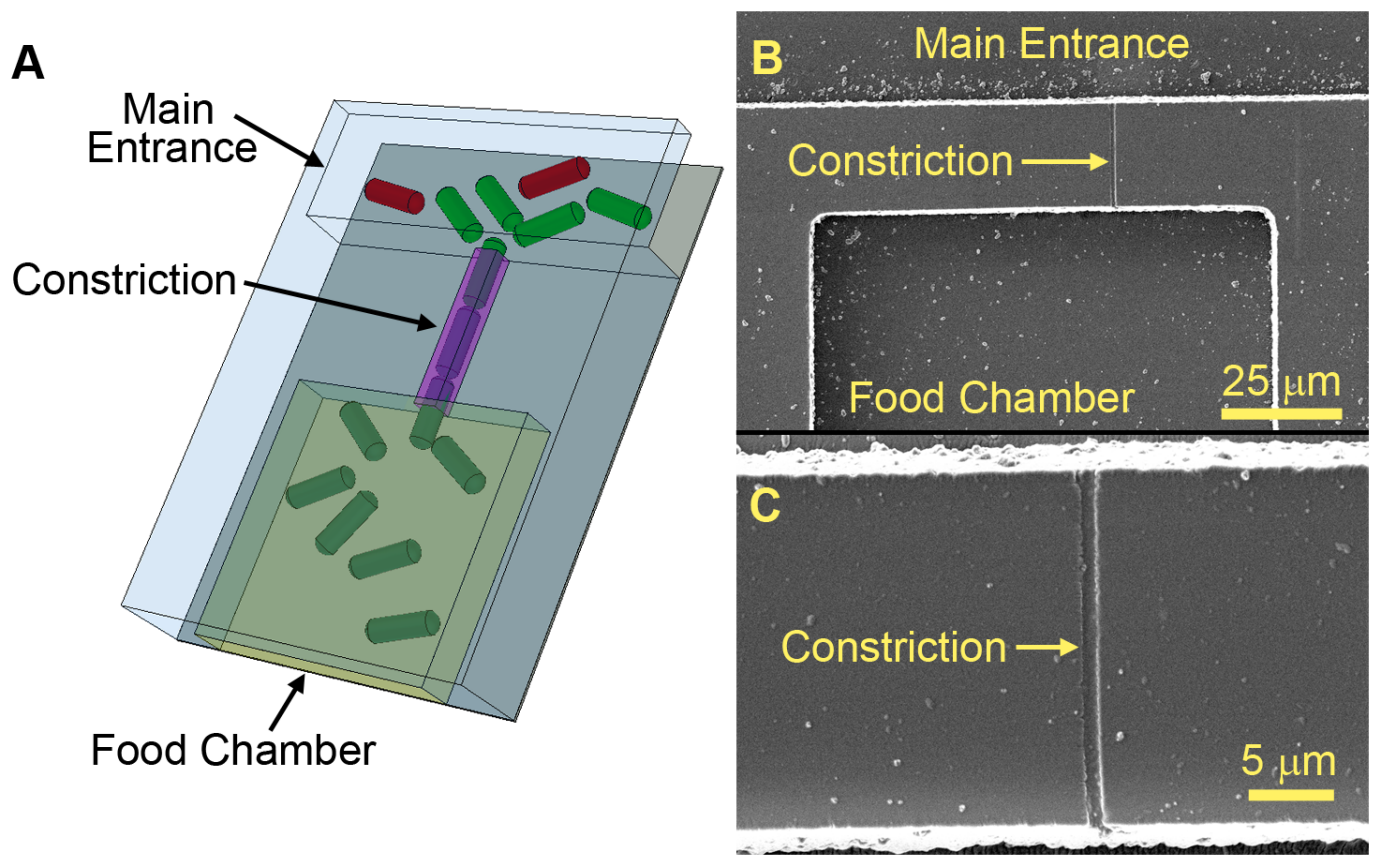
Automated isolation of pure bacterial cultures from the environment. (a) Schematic of one microfluidic device showing part of a single food chambers. The constriction geometry can be varied to isolate species with particular physical characteristics. (b) Scanning electron microscope image of one food chamber that is connected to the outside environment with a sub-micrometer constriction. (c) Magnified image of the constriction shown in (b). (b–c) White particles seen on the SEM images formed during the metal sputtering step of SEM sample preparation. Freshly made polymer chips do not have visible particles on the surface.

A schematic of the operating principle is shown in Figure 1a. Initially, cells sense and move toward nutrients, and other chemoattractants, that are diffusing from large food chambers. The first cell to fit inside the constriction, blocks the entrance to the food chamber, and prevents other cells from entering. The cell can continue to grow and divide, thus populating the food chamber with a single species. The constriction dimensions and nutrient composition in the food chamber can be varied to increase the diversity of captured cells or to optimize the isolation of species with particular characteristics of interest. Though several techniques have merged micro/nano-technology and microbiology to manipulate single cells, the central purpose of our device, and its future application to environmental samples, is unique [27]–[32]. The results presented here are a critical first step toward the creation of a new device for isolation and cultivation of bacterial species.

## Materials and Methods

### Design and Fabrication of the Master Wafer

Constrictions and large channels were designed as separate mask layers using Tanner L-Edit (version 15.1) software. The width and length of each constriction was varied in the designs to determine the optimum conditions for capturing bacteria. 950 nm tall constrictions have widths ranging from 1.5 µm to 5 µm and lengths from 20 µm to 40 µm. The widths of 700 nm tall constrictions are between 0.75 µm and 1.5 µm, while the lengths are between 20 µm and 40 µm. Food chambers are 100–200 µm wide, 7.5 µm tall, and 2–4 mm long while the main channel is 100 µm wide, 7.5 µm tall, and 19 mm long. These dimensions are large compared to the size of bacterial cells and provide adequate space for normal cellular functions such as swimming and growth. A COMSOL multiphysics simulation confirmed that a substantial nutrient gradient exists throughout the course of a multi-day experiment and provides incentive for bacteria to enter the chamber (Figure S1 in File S1).

First, poly (methyl methacrylate) resist (PMMA 950 A9, MicroChem) was spun on a clean 3–inch silicon wafer (University Wafers) at 3000 rpm for 1 minute (Laurell Spinner) and baked at 175°C for 5 minutes. The thickness of the resist was confirmed with an optical profilometer (Nano-Spec) to be 1.3 µm. The constriction design was defined in the PMMA using a dedicated electron-beam writer (Elionix F-125) at 125 kV with a current of 10 nA and dose of 1800 µC/cm^2^. After exposure, the wafer was developed in a 3∶1 (v/v) ratio of isopropanol to methyl isobutyl ketone (MIBK) for 90 seconds and rinsed thoroughly with isopropanol. Chromium metal sputtering was achieved at 500 W and 275 DC Volts (MRC 8667 Sputtering) followed by lift-off with hot acetone to leave only the defined metal constrictions on the wafer. The thickness of the metal on the wafer was confirmed with a surface profiler instrument (DEKTAK Profilometer 3ST). Next, positive photoresist, AZ4620, was spun at 3000 rpm for 1 minute (Laurell Spinner) and baked for 1 minute at 115°C. The food chamber and main entrance channel mask (4″ x 4″ chrome, Front Range Photomask) was aligned over the metal constrictions on the master wafer and the resist was exposed to UV light (Quintel 4000 Mask Aligner). The features were developed in a 3∶1 (v/v) ratio of deionized water to AZ400K developer. At the end of this step, a complete master wafer was obtained.

### Fabrication of Devices

To create patterned poly-dimethyl siloxane (PDMS) polymer microfluidic devices from the master wafer, base and the curing agent were mixed together in a 10∶1 (w/w) ratio (Sylgard 184 Silicone Elastomer Kit, Dow Corning). The patterned master wafer was vapor coated with chlorotrimethylsilane (CTMS) (Acros Organics) prior to pouring the PDMS mixture onto it to allow easy separation of the polymer from the master after curing. The mixture was poured onto the wafer, degassed in vacuum chamber for several minutes to remove any air bubbles, and heat-cured at 70°C for 3 hours. Cured PDMS was peeled from the master and cut into sections, where each section had one device containing multiple food chambers. Scanning Electron Microscope (SEM) images of the master wafer and several PDMS devices were taken with Hitachi S-4800 Field Emission Scanning Electron Microscope. Prior to being imaged in the SEM, the PDMS pieces were sputter coated with a thin layer of Pt/Pd alloy using a Cressington Sputter Coater 208HR.

**Figure 2:**
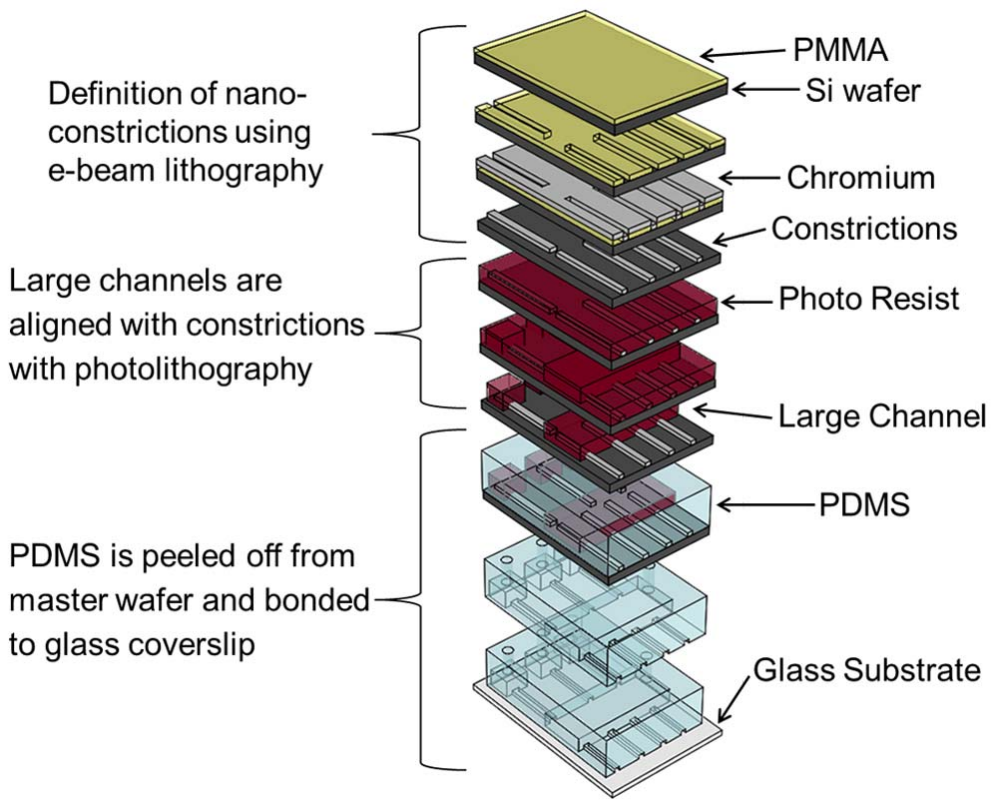
Schematic depicting the device fabrication process. 1) PMMA is spun on a 3-inch silicon wafer. 2) The constriction design is written with electron beam lithography and developed. 3) Chromium is sputtered (the height of the constrictions is determined by this step). 4) Metal lift-off is done to complete the constriction. 5) Positive photoresist is spun on the wafer. 6) Large features are aligned with the constrictions and exposed using photo-lithography. 7) Large features are developed, completing the master wafer. 8) PDMS is poured over the master and heat cured. 9) PDMS is peeled off and access holes are drilled. 10) The PDMS is bonded to a glass cover slip using oxygen plasma to complete the device.

Access holes to food chambers and to the main entrance channel were drilled manually using a sharpened 18G needle (Small Parts). The patterned side of the PDMS was permanently bonded to a microscope cover slip (Fisher Scientific, microscope cover glass, 0.17–0.25 mm thick) using oxygen plasma (Anatech SP-100 Plasma System) at 100 W, 0.4 Torr for 5 sec. A schematic of the device fabrication protocol is shown in Figure 2.

### Bacterial Cultures

M-cherry *Escherichia coli* DH5α strain was cloned with a pUC19 plasmid at high copy number while green fluorescent protein (GFP)-labeled *Escherichia coli* DH5-α strain contains a high copy number of pLIT 29 plasmid. M-cherry is a type of red fluorescent protein (RFP). Both strains were kindly supplied by the Godoy Lab, Northeastern University-Department of Biology. A lac promoter is present in both plasmids and regulates expression of the fluorescent protein genes. An ampicillin resistance gene was inserted into the plasmids as well to ensure the selection of fluorescent protein labeled bacteria from agar plates containing ampicillin (BD, Difco LB, Miller and BD, Difco agar, Technical). Cyan fluorescent protein (CFP)-labeled wild-type *Pseudomonas aeruginosa* PA01 strain, and marine species *Roseobacter sp*. and *Psychroserpens sp*. were supplied by the Epstein Lab, Northeastern University-Department of Biology. *P. aeruginosa* and marine species were cultured in LB-agar plates at 37°C and at room temperature, respectively.

Prior to loading the device with bacteria, both *E. coli* strains were cultured overnight at 37°C in 6 mL of lysogeny broth (LB), which had ampicillin (Sigma Aldrich, ready-made ampicillin) added in at a 1∶1000 (v/v) ratio. The *P. aeruginosa* strain was cultured overnight at 37°C in 6 mL of LB. *Roseobacter sp*. and *Psychroserpens sp*. were cultured at room temperature for 2 days in 6 mL of LB.

### Experimental Procedure and Fluorescence Imaging

Food chambers were filled with LB through the access holes using a 19 Gauge needle connected with tubing to a filled syringe mounted in a syringe pump. The main entrance channel was filled with Phosphate Buffered Saline (PBS) using the same approach. Filling of the device was monitored by eye.

Two bacterial strains were cultured simultaneously in separate vials of growth media, and the cellular concentration of each was determined using a hemocytometer. The cellular concentration was adjusted individually for each species, using phosphate buffer solution, to obtain the target cell ratio at the start of each experiment. Next, 1∶1 (v/v) aliquots of the two adjusted bacterial cultures were collected and thoroughly mixed. A droplet of culture was inoculated into the main entrance channel. The device was then placed in a high humidity chamber and incubated overnight at 37°C. The device containing marine species was incubated at room temperature.

All images were taken with a Zeiss Axio Imager A2 fluorescence microscope at 40X and at 100X using an oil immersion lens with a 1.3 numerical aperture objective. The microscope contained both GFP and RFP filters for fluorescent imaging. Incident light was provided by an X-Cite Series 120Q excitation light source. Zeiss Axiovision (version 4.8.2.0) imaging software was used to process images obtained with an AxioCam MRm CCD camera.

## Results and Discussion

### Fabrication of Devices

Techniques to capture individual bacterial cells in the literature include droplets, lobster traps, and micro-sieves [27], [33], [34]. The devices used in these techniques typically have significant power requirements and require continuous monitoring, which limits their ability to be mass-produced and deployed into the environment. Microfabricated devices overcome these limitations by providing automated handling, separation, and even identification of microorganisms [28], [29], [35]–[38].

We utilized a simple, yet very effective device fabrication strategy to address the need for high-throughput bacterial sample collection and cultivation. Polymer chips containing microscale features were created using replica molding (soft lithography) and bonded onto standard glass microscope cover slips, allowing several devices (and correspondingly dozens of chambers) to be fabricated simultaneously. The replica molding process can be repeated tens of times using a single master to generate large quantities of devices. A scanning electron micrograph (SEM) of one metal-coated food chamber and constriction is shown in Figure 1b. Figure 1c is a magnified view of the constriction connecting the nutrient chamber to the external environment. An SEM of the master used to create the polymer structures is provided in Figure S2 in File S1. While our designs appear to be similar to other microfluidic devices used for microbial research, the critical distinguishing features are the precisely fabricated constrictions with varied sub-micron dimensions. The constrictions coupled with food isolation chambers allow capture of individual bacterial cells from heterogeneous samples and provide single species cultures.

### Inter-Species Separation

To demonstrate the functionality of our method, we first separated mixtures of two bacterial species commonly found in nature: *Escherichia coli* and *Pseudomonas aeruginosa*. Critical to the successful isolation of individual species are the dimensions of the constriction. To impede the movement of bacterial cells through the constriction, at least one dimension of the constriction cross-section must be smaller than the smallest dimension of the cell [37]. The diameters of *E. coli* and *P. aeruginosa* are approximately 1.0 µm and 0.8 µm, respectively [39], [40]. We therefore fabricated devices with constriction heights of 700 and 950 nm.

In the first set of experiments, CFP-labeled *P. aeruginosa* and RFP-labeled *E. coli* from liquid cultures were mixed in a 1∶1 (v/v) ratio to obtain a mixed sample. Each culture contained a similar cell density, which was verified using hemocytometer counts. To determine the optimal separation conditions, devices were tested with 700 nm high constrictions that had widths ranging from 0.75 to 1.50 µm and lengths from 10 to 40 µm. The 0.75 µm width constrictions were smaller than the average cross-sectional area of both bacterial species and the largest width of 1.50 µm created a cross-sectional area larger than that of both species. Different constriction channel lengths were tested to determine if this parameter would affect separation as well.

### Effects of Constriction Design and Initial Concentration of Microorganisms

**Figure 3:**
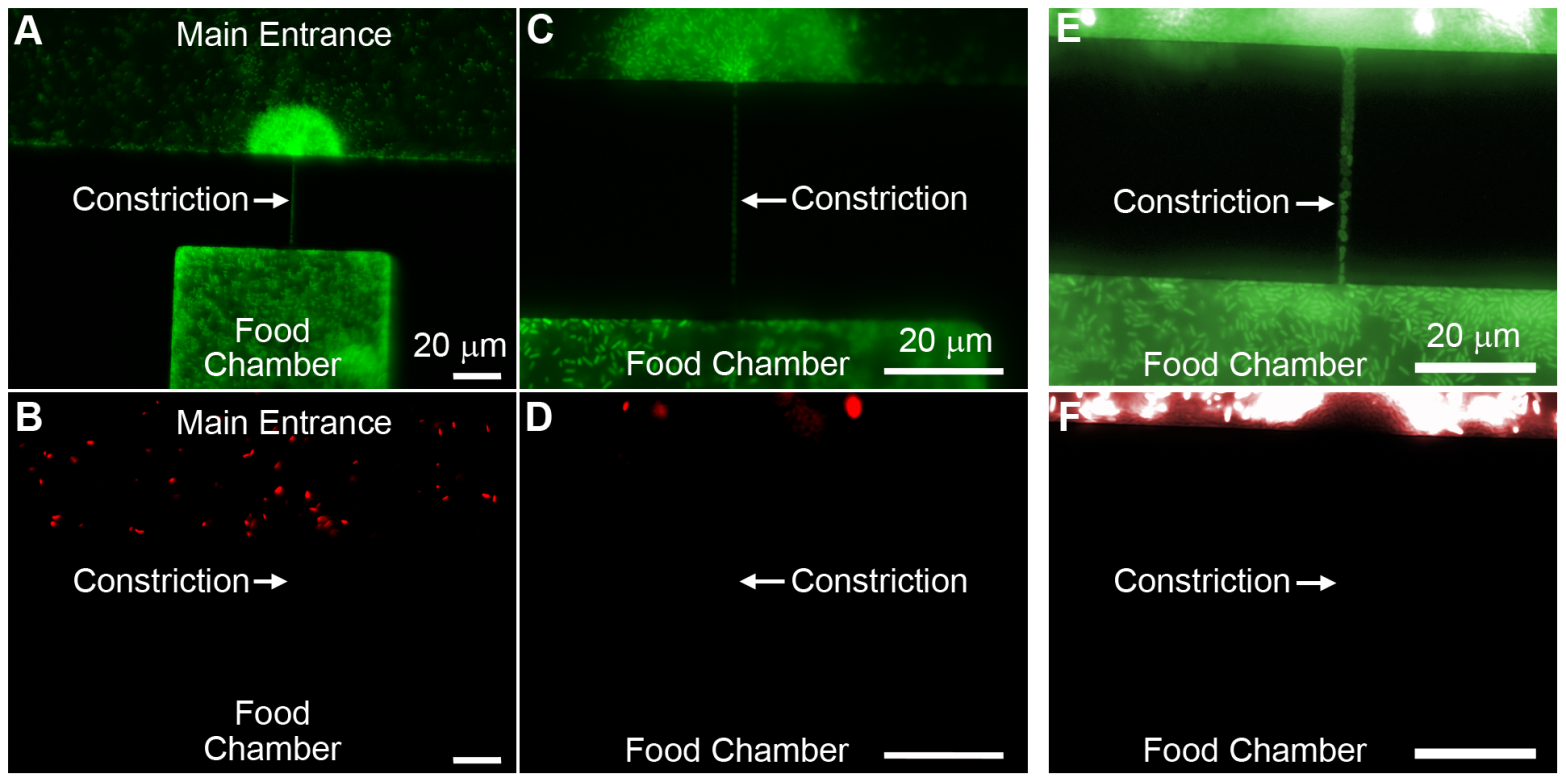
Isolation of pure bacterial cultures from heterogeneous populations. (a-d) Food chamber connected to an external environment containing CFP-labeled *P. aeruginosa* and RFP-labeled *E. coli* via a 700 nm tall, 1.5 µm wide, 40 µm long constriction. (a) GFP filter, 40x magnification. (b) RFP filter, 40x magnification. *E. coli* are only observed in the main entrance. (c) GFP filter, 100x magnification. A single column of *P. aeruginosa* cells is in the constriction. (d) RFP filter, 100x magnification. (e-f) Food chamber connected to an external environment containing GFP-labeled and RFP-labeled *E. coli* via a 950 nm tall, 1.5 µm wide, 40 µm long constriction. (e) GFP filter, 100x magnification. A single column of GFP-labeled cells is visible. (f) RFP filter, 100x magnification. No RFP-labeled cells are visible in the constriction or food chamber.

Figure 3a and 3c show GFP filtered images of a 700 nm high, 1.5 µm wide constriction connected to a food chamber at 40x and 100x magnification, respectively, after inoculation with both species and overnight incubation at 37°C. Both images clearly illustrate the chemotactic behavior of bacteria toward the food chamber and biofilm formation at the entrance of the constriction. A single file line of CFP-labeled *P. aeruginosa* cells is seen in the constriction in Figure 3c. Figure 3b and 3d are RFP filtered images of the same constriction and chamber at 40x and 100x magnification, respectively. These images confirm that both species were present in the environment outside the chamber, while only CFP-labeled *P. aeruginosa* entered the constriction.

Deformation of bacterial shape did not noticeably alter the viability or motility of the cells, as images collected after cells crossed the constriction always showed *P. aeruginosa* proliferating and swimming inside the food chamber in the same manner as in the main chamber. Figure 3e shows individual deformed cells in the constriction and normal-shaped cells in the food chamber. Interestingly, although both species exhibited a chemotactic response and formed biofilms at the entrance of every constriction, neither *P. aeruginosa* nor *E. coli* entered constrictions that were less than 1.5 µm in width (Figure S3 in File S1). *E. coli* have been reported to enter constrictions that are less than 700 nm high in other experiments [37], which raises questions regarding the importance of the chemical environment and phenotypic differences between strains. The length of constrictions did not affect species isolation. To determine the effects of starting concentration on the isolation of species, experiments with more *E. coli* cells in the initial inoculum having a 2∶1 and 1000∶1 (v/v) ratio were also run. The results from these experiments are shown in Figures S6 and S7, respectively, in File S1. Even though more *E. coli* cells were present initially, the *P. aeruginosa* population always outgrew the other species. This is because *P. aeruginosa* produces pyocyanin and other toxins that act as antibiotics against competing bacterial species [41].

### Separation Efficiency of Devices

The experiments were repeated multiple times using devices that had the same range of constriction widths and lengths, but the height was 950 nm. The results show that the isolation of *P. aeruginosa* from *E. coli* was again successful (Figure S4 in File S1). As the width of the constriction is increased beyond 2.0 µm, the bacteria start to form multiple lines along the channel (Figure S5 in File S1). The success rate of isolating individual species in food chambers also decreased with increasing constriction widths. In total, 49 constrictions with widths varying from 1.5 µm to 5 µm, and with a height of 950 nm were tested using a mixture of *P. aeruginosa* and *E. coli* (Table 1). A single species was observed in 22 of the 49 food chambers. 11 of 21 constrictions with widths ranging from 1.5 µm to 2 µm had cells arranged in single file, as shown in Figure 3, and Figure S4 in File S1, and only contained one species in their food chambers. 10 of the 21 constrictions in this width range had no growth in the constriction. The results for these 21 constrictions are ideal as all of the food chambers contained 1 or 0 species.

**Table 1:** Separation efficiency data of devices: 49 constrictions with various widths were used to separate a mixture of CFP *P. aeruginosa* and m-cherry *E. coli*.

The width of 950 nm tall constrictions (µm)	Number of constrictions tested	Food chambers with single species: Single line in constriction	Food chambers with single species: Multiple lines in constriction	Food chambers with multiple species	Food chambers with no cell growth
**1.5**	7	5	-	-	2
**1.75**	7	2	-	-	5
**2**	7	4	-	-	3
**2.5**	7	-	2	1	4
**3**	7	-	3	1	3
**4**	7	-	3	1	3
**5**	7	-	3	4	-
**Total**	**49**	**11**	**11**	**7**	**20**

All of the constrictions were 950 nm tall.

28 constrictions were tested that had a width 2.5 µm or greater. 11 of these constrictions had multiple lines of cells, but still contained a single species in the food chamber, similar to the results shown in Figure S5 in File S1. 7 food chambers connected to these constrictions contained both species while 10 remained empty. The separation efficiency decreases for constrictions having width 2.5 µm or greater, but is still quite good at 39%.

Of the 20 chambers that did not have cells grow inside, 16 had constrictions that were blocked by dust or air bubbles, which prevented cells from entering. Compared against other currently used environmental culture isolation techniques, this separation efficiency is very promising. 20 blocked or unfilled constrictions out of 49 in our PDMS prototypes is a manageable amount that will not preclude the devices from functioning in practical applications. We are however investigating ways to minimize losses due to air bubbles by using materials that are more hydrophilic than PDMS to construct the devices. Further, hundreds of constrictions will be utilized in complex environments to account for unexpected blockages.

The isolated individual species were removed from the device, for further analysis and culturing, using a syringe. We removed the separated CFP *P. aeruginosa* cells from the food chambers and spread plated them onto lysogeny broth (LB) growth medium agar plates. After overnight incubation, the plates where inspected under a microscope and only cyan colored colonies were observed, further confirming successful isolation of an individual species.

### Intra-Species Separation

To confirm that a single cell is sufficient to block the constriction, we completed a second set of experiments using only GFP-labeled and RFP-labeled strains of *E. coli*. Devices containing 700 nm or 950 nm high constrictions were inoculated with the two strains and incubated overnight at 37°C. Neither strain of *E. coli* was able to enter the 700 nm tall constrictions. This result is also in correlation with the images shown in Figure 3b and 3d. *E. coli* successfully entered food chambers connected to the external environment via 950 nm high, 1.5 µm wide and wider constrictions. No cells were observed in food chambers connected to narrower constrictions. Importantly, only one color of cells was present in any given chamber, supporting the hypothesis that the cells in a food chamber are daughters of a single cell in the constriction. Figure 3e and 3f demonstrate the successful isolation of GFP-labeled *E. coli* from a mixture containing both GFP- and RFP-labeled cells. Also seen in Figures 3e and 3f is a biofilm of GFP-labeled *E. coli* around the constriction entrance. While some progeny of the initial green cell are dividing across the constriction, other daughter cells are growing and dividing at the entrance of the constriction to form a biofilm, which may also aid in preventing red cells from reaching the constriction.

### Marine-Species Separation

**Figure 4:**
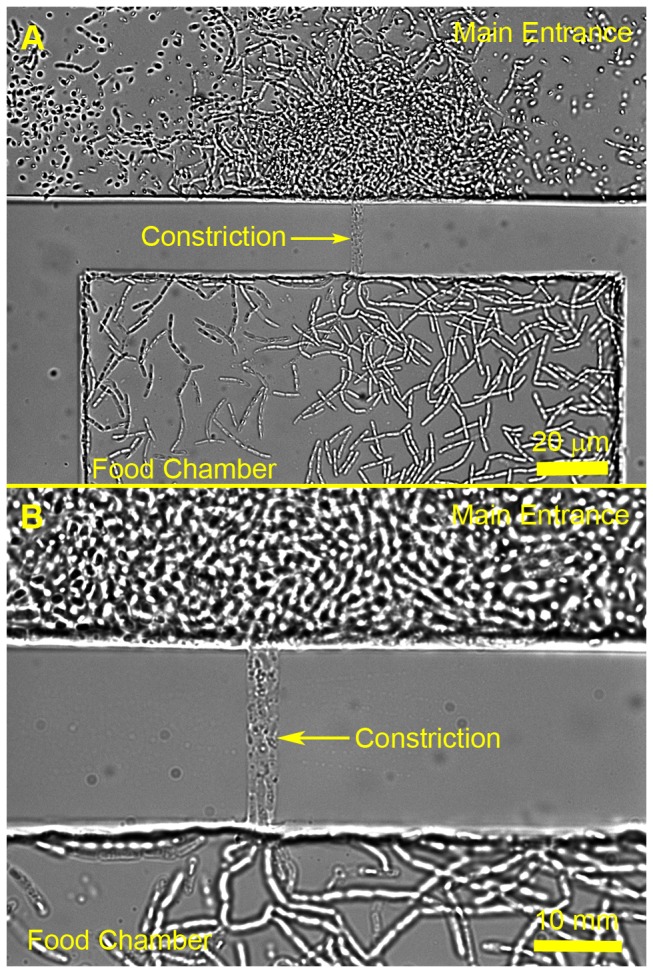
Isolation of *Roseobacter sp*. from *Psychroserpens sp*. (a-b) Food chamber connected to main entrance containing heterogeneous bacterial sample via a 950 nm tall, 5 µm wide, and 30 µm long constriction. (a) Brightfield, 40x magnification. (b) Brightfield, 100x magnification. Both species are present in the main entrance. Only *Roseobacter sp*. cells are present in the constriction and food chamber.

Our next step was to demonstrate that our devices can be applied for the separation of environmental microorganisms. For this purpose, we selected two marine species that were recently collected from the environment: *Psychroserpens sp*. and *Roseobacter sp*., which are cocci- and rod-shaped bacteria, respectively. Devices with 950 nm high constrictions were used for this set of experiments. Both species grew well in the main entrance of devices. Even though bacteria were not labeled with fluorescent protein genes for easy detection, the presence of both species in the device can be simply distinguished by the difference in their morphology, as seen in Figure 4a. Figure 4 (a, b) shows a food chamber connected to the main entrance with a 5 µm wide constriction. Figure 4b, which is a 100x magnified image of the constriction and the food chamber, shows that only *Roseobacter sp*. is present in the constriction and food chamber since only rod-shaped cells can be seen in the food chamber. A sample was removed from the food chamber and cultured on LB-agar plates to confirm that *Roseobacter sp*. was successfully isolated. Interestingly, 5 µm wide constrictions showed multiple lines of cells, but narrower constrictions did not show any bacterial growth. This result suggests that a different or wider range of constriction sizes may be necessary for isolating and separating other species in the environment. This result also raises many questions regarding the behavior of bacteria in confined spaces. Further investigations are being completed to determine the cause of this behavior.

### Culturing isolated species

There is a risk of contaminating the isolated pure cultures when they are removed from the food chamber. To prevent possible contamination, the access holes of food chambers are located several millimeters away from the main entrance where the constriction is located. The exterior of the device, particularly the area around the food chamber, is sterilized with 70% ethanol prior to the recovery of species.

In these experiments, each collected sample was spread plated on LB-agar solid media and the fluorescence/morphology of the resulting colonies was evaluated to confirm that contamination did not occur during the removal process. Several samples were also randomly taken from these plates, resuspended in liquid growth media, and observed under a microscope. Both of these results showed the growth of a single species. Figure S8 in File S1 shows the culture plate and subsequently resuspended *Roseobacter sp*. cells that were successfully isolated from *Psychroserpens sp*. using our device.

## Conclusions

When attempting to isolate a specific known species using the described method, phenotypic differences between species must be exploited. While constriction size plays an important role, for targeted isolation and discovery other physiological factors may need to be considered as well. For example, *P. aeruginosa* are much faster swimmers than *E. coli* causing them to reach constrictions much more rapidly. Species that grow rapidly or secrete toxic molecules also have a selective advantage. The proposed method can be tailored and adapted to minimize these differences by changing nutrient composition in the food chamber and the environmental conditions in the immediate vicinity of the constriction entrance. It is also noteworthy to mention that both *P. aeruginosa* and *E. coli* cells rapidly form a biofilm around the constriction entrance, which potentially helps prevent the other species from reaching the constriction and food chamber. The constrictions can also be used, in the future, to allow complete chemical communication while maintaining spatial separation of species and strains of cells.

We demonstrated isolation of cells from mixtures containing two cell types, but the concept applies to systems of any complexity. The results presented here validate the ability of our completely passive devices to isolate individual bacterial species from heterogeneous populations and supports the use of this technique for high-throughput screening of new and/or mutated microorganisms in virtually any environment. By increasing the number of food chambers with constrictions, the device can be scaled to isolate many different individual species and strains from mixtures containing a large microbial diversity where growth conditions are unknown. By employing hundreds of such devices in the next step of this project, we expect to isolate uncultivated species from the environment.

## Supporting Information

File S1 PDF file containing Figures S1–S8.
(PDF)
