# Supplementary material for: Isolation of Microorganisms Using Sub-Micrometer Constrictions
Source: PLoS One. 2014 Jun 30;9(6):e101429. doi: 10.1371/journal.pone.0101429 (PMC4076310; doi:10.1371/journal.pone.0101429)
Supplement: File S1 — PDF file containing Figures S1–S8. (PDF) [file pone.0101429.s001.pdf]

File S1. SUPPLEMENTARY INFORMATION

Isolation of Microorganisms using Sub-Micrometer Constrictions

Nil Tandogan,[a] Pegah N. Abadian,[a] Slava Epstein,[*b] Yoshiteru Aoi[*b,c] and Edgar D. Goluch*[a]

[a] Department of Chemical Engineering, Northeastern University, Boston MA 02115, U.S.A.

[b] Department of Biology, Northeastern University, Boston, MA 02115, U.S.A.

[c] Institute for Sustainable Science and Development, Hiroshima University, Hiroshima 739-8527, Japan

* Corresponding Authors: email: e.goluch@neu.edu, telephone: +1-617-373-3500; yoshiteruaoi@hiroshima-u.ac.jp; s.epstein@neu.edu

Computational Modeling of the Device

The diffusion rate of nutrients is critical to attract bacteria towards constrictions. We modeled the device performance using COMSOL 4.2 multiphysics software. The modeled design contained a single 7 µm tall, 200 µm wide, 4 mm long food chamber initially containing 8.8 mM leucine connected to a large reservoir of pure water via a 1 µm tall, 1.5 µm wide, 40 µm long constriction. Leucine is one of the primary amino acid present in LB growth medium [1]. The concentration of leucine in the food chamber was plotted versus time, depicted in Supplementary Figure 1.

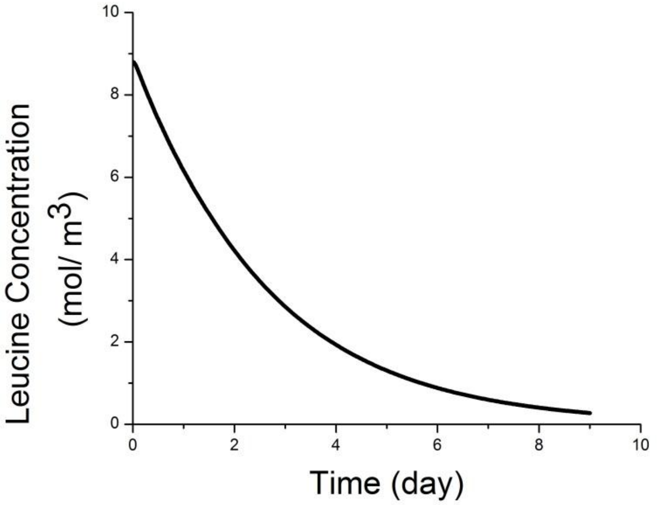

Figure S1. Computational model showing the concentration of nutrients versus time in a food chamber that is connected to the outside environment by a nano-constriction.

Additional Data

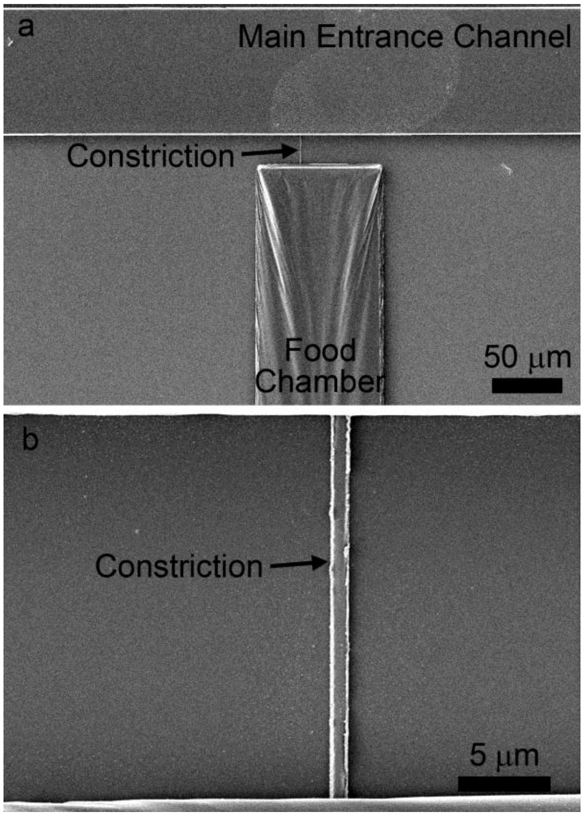

Figure S2. (a, b) Scanning electron microscope images of a patterned master wafer. Constrictions were fabricated with electron-beam lithography to obtain exact widths at high precision in a single step. The constriction is made from chromium metal. The main channel and food chamber are made with AZ 4620 photoresist. The master was sputter coated with Pt/Pd prior to being placed in the SEM.

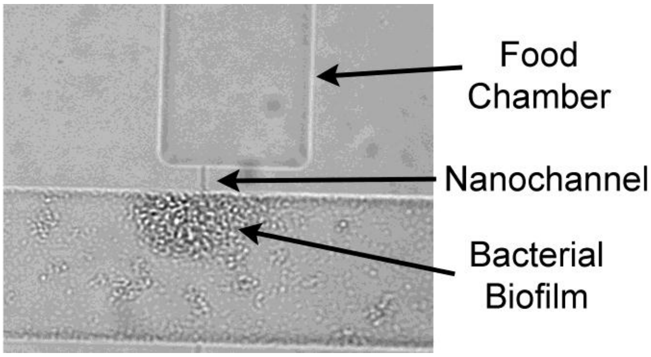

Figure S3. Optical micrograph image of bacteria forming a biofilm at the entrance of a nanochannel connecting two microchannels made from PDMS. The bacteria are attracted to the fresh growth media diffusing through the nanochannel, but cannot squeeze through to get to the food source. Adapted from [2].

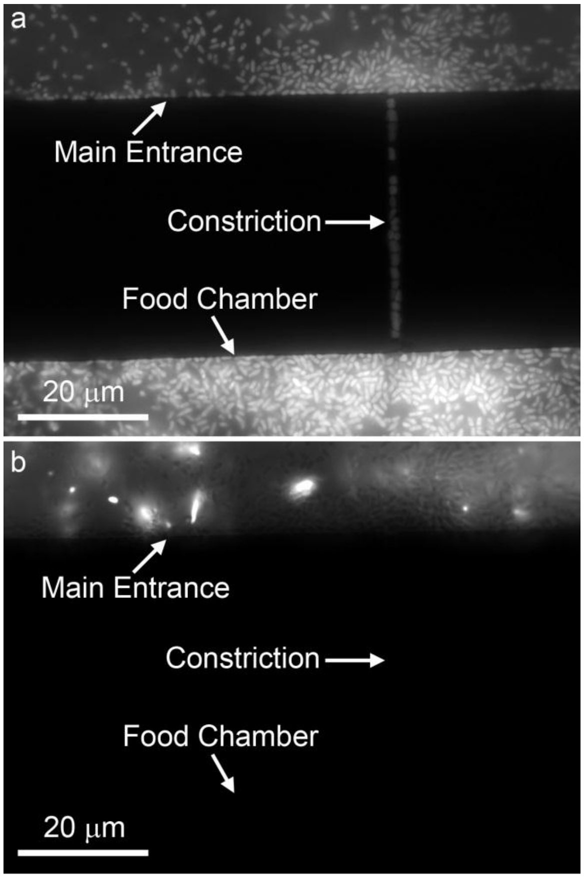

Figure S4. (a, b) GFP and RFP filtered images of a device containing a 950 nm tall, 2 µm wide, and 40 µm long constriction that was inoculated with CFP *P. aeruginosa* PA01 and m-cherry *E. coli*. (**a)** GFP filtered image of *P. aeruginosa* forming a single line in the constriction and entering the food chamber. (**b**) RFP filtered image of the same constriction and food chamber, proving that *E. coli* is located solely in the main channel.

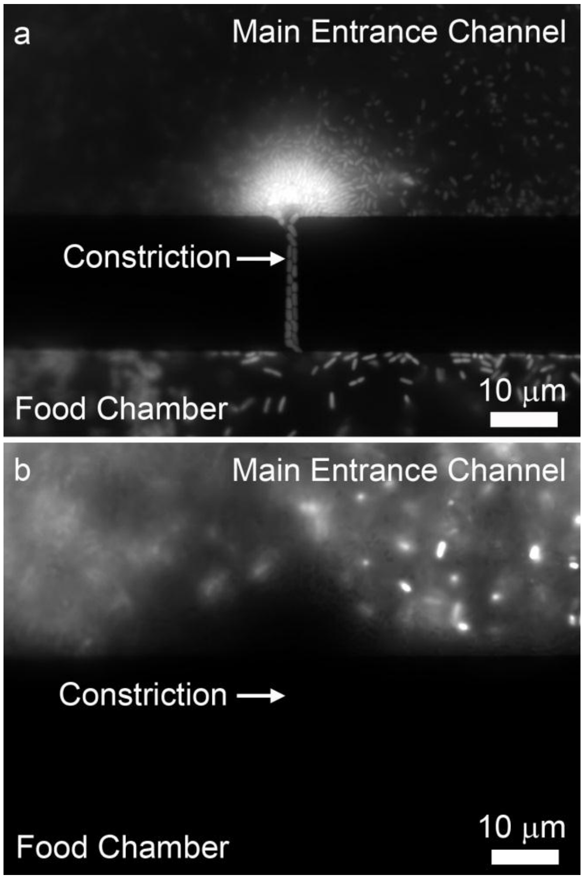

Figure S5. Isolation of CFP *P. aeruginosa* through wide constrictions. (**a)** GFP filtered image. CFP *P. aeruginosa* formed 2 lines inside the constriction. (**b)** RFP filtered image. m-cherry *E. coli* did not grow in the constriction or food chamber that was blocked by *P. aeruginosa*. The constriction is 950 nm tall, 2.5 µm wide and 20 µm long.

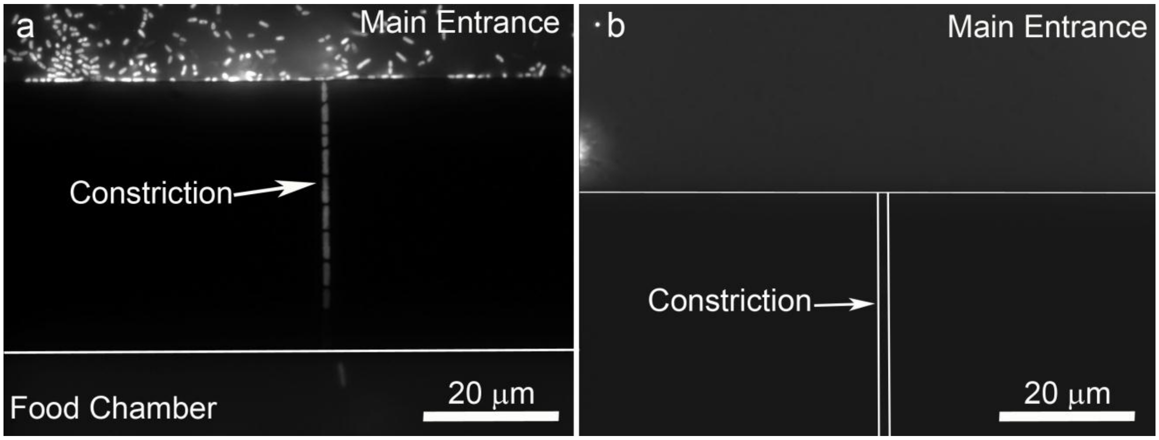

Figure S6. (a) GFP and **(b)** RFP filtered images of 2:1 (v/v and concentration) ratio of m-cherry *E. coli*: CFP *P. aeruginosa* in a device with 700 nm tall, 1.5 µm wide constriction respectively. **(a)** CFP *P. aeruginosa* entered the food channel by forming a visible single file in the constriction **(b)** m-cherry *E. coli* were present in the main channel, but their concentration was much lower than *P. aeruginosa* even though their initial inoculum concentration was higher than *P. aeruginosa*.

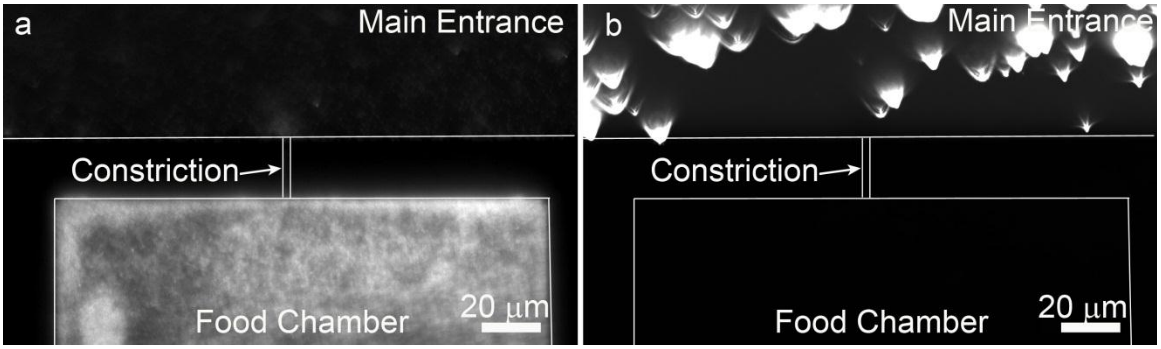

Figure S7. (a) GFP and **(b)** RFP filtered images of 1000:1 (v/v and concentration) m-cherry *E. coli* : CFP *P. aeruginosa* in a device with 950 nm tall, 1.75 µm wide constrictions. **(a)** CFP *P. aeruginosa* entered and populated the food chamber preventing *E. coli* from entering. *P. aeruginosa* in the main channel and in the constriction were not visible using fluorescence microscopy due to photobleaching. **(b)** m-cherry *E. coli* were present in the main channel, but could not enter the food channel.

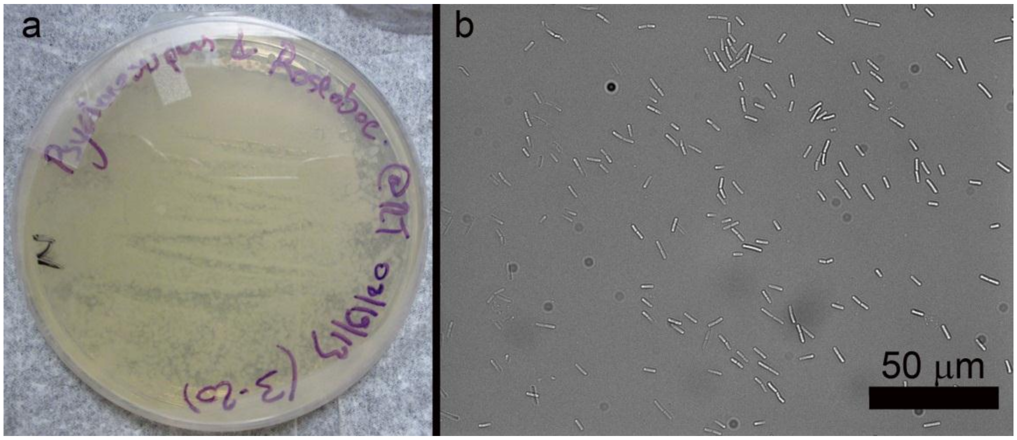

Figure S8. **(a)** Photograph of a culture plate containing only *Roseobacter sp.* colonies. The sample was collected from the food chamber shown in Figure 4 and spread on the plate. **(b)** Micrograph of individual *Roseobacter sp*. cells swimming in liquid growth media that were cultured from the plate shown in (a). No *Psychroserpens sp.* are visible in either image.

References

1. Sezonov G, Joseleau-Petit D, D'Ari R (2007) *J Bacteriol* 189, 8746-8749.
2. Abadian PN, Tandogan N, Webster TA, Goluch ED (2012) *microTAS 2012* 413-415.
